# EGR1 Functions as a Potent Repressor of MEF2 Transcriptional Activity

**DOI:** 10.1371/journal.pone.0127641

**Published:** 2015-05-26

**Authors:** Yi Feng, Cody A. Desjardins, Olivia Cooper, Akuah Kontor, Sarah E. Nocco, Francisco J. Naya

**Affiliations:** Department of Biology, Program in Cell and Molecular Biology, Boston University, Boston, Massachusetts, United States of America; Cincinnati Children's Medical Center, UNITED STATES

## Abstract

The myocyte enhancer factor 2 (MEF2) transcription factor requires interactions with co-factors for precise regulation of its target genes. Our lab previously reported that the mammalian MEF2A isoform regulates the cardiomyocyte costamere, a critical muscle-specific focal adhesion complex involved in contractility, through its transcriptional control of genes encoding proteins localized to this cytoskeletal structure. To further dissect the transcriptional mechanisms of costamere gene regulation and identify potential co-regulators of MEF2A, a bioinformatics analysis of transcription factor binding sites was performed using the proximal promoter regions of selected costamere genes. One of these predicted sites belongs to the early growth response (EGR) transcription factor family. The EGR1 isoform has been shown to be involved in a number of pathways in cardiovascular homeostasis and disease, making it an intriguing candidate MEF2 coregulator to further characterize. Here, we demonstrate that EGR1 interacts with MEF2A and is a potent and specific repressor of MEF2 transcriptional activity. Furthermore, we show that costamere gene expression in cardiomyocytes is dependent on EGR1 transcriptional activity. This study identifies a mechanism by which MEF2 activity can be modulated to ensure that costamere gene expression is maintained at levels commensurate with cardiomyocyte contractile activity.

## Introduction

Members of the myocyte enhancer factor 2 (MEF2) family of transcription factors play essential and diverse roles in tissue development and function as exemplified by mutant phenotypes in mice and other animal model systems [1]. The transcriptional function of MEF2 is primarily modulated through signaling pathways and interactions with coregulators that can either enhance or abrogate its activity in specific biological settings [2]. While this notion is firmly established, considerably less is known about the mechanism(s) by which MEF2 coordinately regulates defined gene programs in muscle.

We have previously reported that cardiomyocyte cytoarchitecture and survival is dependent on MEF2A [3,4]. MEF2A was shown to modulate the integrity of the cardiomyocyte cytoskeleton through its direct regulation of a collection of genes encoding proteins localized to the costamere, a muscle-specific focal adhesion which connects the myofibrils to the plasma membrane (sarcolemma) and functions to transmit contractile forces throughout the myocyte [4–6]. To gain further insight into the mechanism by which MEF2A regulates a costamere gene program, a bioinformatics analysis of transcription factor binding sites was performed using the proximal promoter regions of costamere genes [4]. This computational approach identified a number of candidate *cis*-elements that may function as binding sites for transcriptional co-regulators of MEF2A-dependent costamere genes. One of these predicted sites belonged to the early growth response (EGR) family of zinc finger transcription factors [7].

The involvement of the EGR1 transcription factor in cardiovascular and neuronal pathways, systems in which MEF2 functions as a central regulator, makes it a particularly attractive candidate coregulatory factor. For example, EGR1 is a downstream effector in atherosclerosis, angiogenesis, and cardiac hypertrophy [8], and has been shown to regulate gene expression in vascular smooth muscle downstream of mechanical stretch [9], a stimulus that modulates costamere/focal adhesion activity. Additionally, like MEF2, EGR1 responds to neuronal activity and regulates expression of genes involved in synapse remodeling [10–13].

In this study, we examined the ability of EGR1 to modulate MEF2A transcriptional activity. We found that EGR1 is a potent repressor of MEF2A transcriptional activity on MEF2-dependent promoters in both non-cardiac and cardiac cells. Consistent with its function as a repressor of MEF2 activity, overexpression and inhibition of EGR1 resulted in down- and up-regulated expression of costamere genes, respectively. Taken together, these results suggest a potential role for EGR1 to modulate MEF2 activity in the regulation of costamere gene expression in cardiomyocytes.

## Materials and Methods

### Cloning of myc-EGR1

EGR1 was PCR-amplified from pcDNA3-Flag-EGR1 (Addgene) using forward primer: 5’-GCAGCGGCCAAGGCCGAGATGCAATT-3’, and reverse primer: 5’-AATAGGGCCCTCTAGATGCATGCTCGAGCGGC-3’. This PCR fragment was subsequently cloned into pcDNA3-myc (N-terminal epitope). Murine MEF2A was PCR amplified from mouse heart cDNA and cloned into pCMV4-Tag (C-terminal FLAG epitope).

### Cell culture, transfection, and luciferase assays

HEK293T embryonic kidney cells were maintained in Dulbecco’s Modified Eagle Medium (DMEM) supplemented with 10% fetal bovine serum (FBS), 1% L-glutamine (L-Glut), and 1% penicillin-streptomycin (pen-strep). HEK293T cells were seeded one day prior to transfection in 6-well plates at a density of 7.5 x 10^4^ per well. Transfections were performed using 0.75 μg of total DNA, with equal amounts of plasmid in each mixture using 1 μg/μL polyethylenimine (PEI) at a 6:1 PEI to DNA ratio. Luciferase experiments were performed on whole cell lysates from cells harvested 36–48 h post transfection. Luciferase readings were normalized by Bradford assay and performed in triplicate, with the exception of the p300-luc experiment, which was normalized to βgal activity.

NRVMs were extracted from ventricles of neonatal rat pups as described previously [4]. Briefly, one to two day old pups were placed on ice for 10 minutes, then decapitated and the hearts were removed and stored in Hank’s Balanced Saline Solution (HBSS). Atria were then removed and ventricles were minced and incubated in 0.06% trypsin solution overnight. Heart fragments were then digested in a Collagenase solution, and cells were preplated twice for one hour each before counting using a hemocytometer and plated on 0.1% gelatin treated cell culture dishes. Twenty-four hours after seeding NRVMs were washed with 1X PBS and subsequently maintained in DMEM containing 0.5x nutridoma, 1% L-glutamine (L-Glut), and 1% penicillin-streptomycin (pen-strep) (Roche), with the exception of NRVMs to be used of siRNA transfections, which were cultured in media lacking 1% L-glutamine (L-Glut), and 1% penicillin-streptomycin (pen-strep). Transfections were performed using 0.75 μg of total DNA, with equal amounts of plasmid in each mixture using 1 μg/μL Fugene6 (Promega) at a 3:1 PEI to DNA ratio. Luciferase experiments were performed on whole cell lysates from cells harvested 36–48 h post transfection. Luciferase readings were normalized to Renilla luciferase internal control readings.

Expression vectors used in this study including pcDNA1-MEF2A, pcDNA3-MEF2A, 3x-MEF2-luc, 1.5kb *myomaxin*-luc, *Tcap*-luc have been described previously [4,14]. *SM22*-luc was a generous gift from Joe Miano (U. Rochester Medical School, NY), and p300-luc was acquired from Addgene.

### Co-immunoprecipitation and western blot analysis

HEK293T cells seeded in 10cm or 6cm plates. Cells were transfected with 10 μg pcDNA3-myc (empty vector) or pcDNA3-myc-EGR1 and 5 μg of pCMV-MEF2A-Flag. 36–48 hours post-transfection, protein was harvested in AT buffer (20% glycerol, 1% Triton X-100, 20 mM HEPES pH7.9, 1 mM EDTA, 150 mM NaCl, 1 mM DTT, 1 μg/mL PMSF, and 1:25 protease inhibitor mixture (Roche). Approximately 35 μL Protein G sepharose beads (GE Healthcare) and 1 μg of anti-Myc was added and incubated with AT buffer precursor (AT buffer excluding DTT, PMSF, and protease inhibitors). The beads, protein, and antibodies were incubated at 4°C, rotating overnight. Samples were boiled samples and loaded onto an 8% SDS-PAGE Gel. A western blot was then run as outlined below, using 1:2,000 anti-Flag as a primary antibody.

Western blots were performed as previously described [15]. Primary antibodies used were as follows: 1:10,000 anti-Flag (Sigma), 1:2,000 anti-c-myc (Santa Cruz Biotechnology), 1:2,000 anti-MEF2 (Santa Cruz Biotechnology), and 1:2,000 anti-GAPDH (Santa Cruz Biotechnology). Blots were incubated with horseradish peroxidase-conjugated secondary antibodies (1:10,000) and reacted with western lighting chemiluminescent reagent (Perkin Elmer) and subsequently exposed to Blue Lite AutoRad film (BioExpress).

### siRNA knockdown in NRVMs and qRT-PCR

Rat EGR1 silencer select siRNA (s127691) and silencer select negative control #1 siRNA were purchased from Invitrogen. These siRNA were resuspended in 500 μL of sterile Baxter water to a final concentration of 10 μM. Media was changed 24 hours post-transfection and total RNA was extracted from the NRVMs 72-hours post transfection by Trizol followed by cDNA synthesis using M-MLV reverse transcriptase (New England Biolabs). Quantitative RT-PCR (qRT-PCR) reactions were run in triplicate for each set of primers and analyzed with ABI 7900 Real Time PCR machine. Primer sequences used for this analysis were previously described in [4].

### Adenoviral amplification, purification, and transduction

AdEGR1 (3.16 x 10^10^ pfu/mL) was kindly given to us by John Davis (University of Nebraska Medical Center). The Adβgal (6.3x10^10^ pfu/mL) served as a negative transduction control and was a kind gift of Ken Walsh (Boston University School of Medicine).

NRVMs were transduced with AdEGR1 or Adβgal at an MOI of 25. The adenovirus stocks were diluted in serum-free DMEM when needed prior to addition to the NRVM cultures. For reporter assays, NRVMs in 6-well plates were transfected with 250 ng luciferase-reporter constructs (including 1.5kb *myomaxin*-luc and 1.5kb *myomaxin* ΔMEF2-luc) and 100 ng tk-Renilla using Trans-IT (Mirus Bio).

NRVMs transduced with AdEGR1 or Adβgal were harvested for RNA, protein, or luciferase assays 72 hours post-transduction. Prior to harvesting RNA, NRVMs were imaged using an Olympus spinning disk confocal microscope. Total RNA was extracted by homogenization by TRizol followed by cDNA synthesis using M-MLV reverse transcriptase (New England Biolabs). Quantitative RT-PCR (qRT-PCR) reactions were run in triplicate for each set of primers and analyzed with ABI 7900 Real Time PCR machine.

### Viability assays

For Cell Titer Blue viability assays, NRVMs were isolated as stated above and seeded onto 24-well plates at densities of 2.5, 5, 10, 20, 40, and 80 thousand cells per well. After 24 hours, recovery media was aspirated and cells subsequently maintained in DMEM containing 0.5x nutridoma (Roche). Twenty four hours later NRVMs were transduced with AdEGR1 or Adβgal. Forty-eight hours post-transduction cell titer blue reagent was added to each well and allowed to incubate for an additional 12 hours. Media from each well was aliquoted onto a 96-well plate and fluorescence was measured using a Victor III microplate reader (Perkin Elmer).

An apoptotic activity assay was also performed by measuring the activity of Caspase 3. 1x10^6^ cells were seeded into 6-well plates, and recovered for 24 hours. The cells were then transduced with AdEGR1 or Adβgal were harvested for protein 72 hours post-transduction. Cell lysates were then incubated with 50μM Ac-DEVD-AMC (BD Pharmigen), a fluorogenic substrate for Caspase 3, for 1 hour at 37°C. The amount of Caspase 3 activity was then measured using a Victor III1420 fluorimeter (Perkin Elmer) with an excitation wavelength of 380nm and an emission wavelength of 460nm. Readings were normalized to Bradford assay for each sample. Assay was run in biological and technical triplicates.

### Ethics statement

Experimental procedures on animals used in this study were reviewed and approved by the Institutional Animal Care and Use Committees (IACUC) of Boston University (protocol number 13–048). These studies were conducted in accordance with the principles of animal care and experimentation in the Guide For the Care and Use of Laboratory Animals.

## Results

### EGR1 potently represses MEF2 transcriptional activity

**Fig 1:**
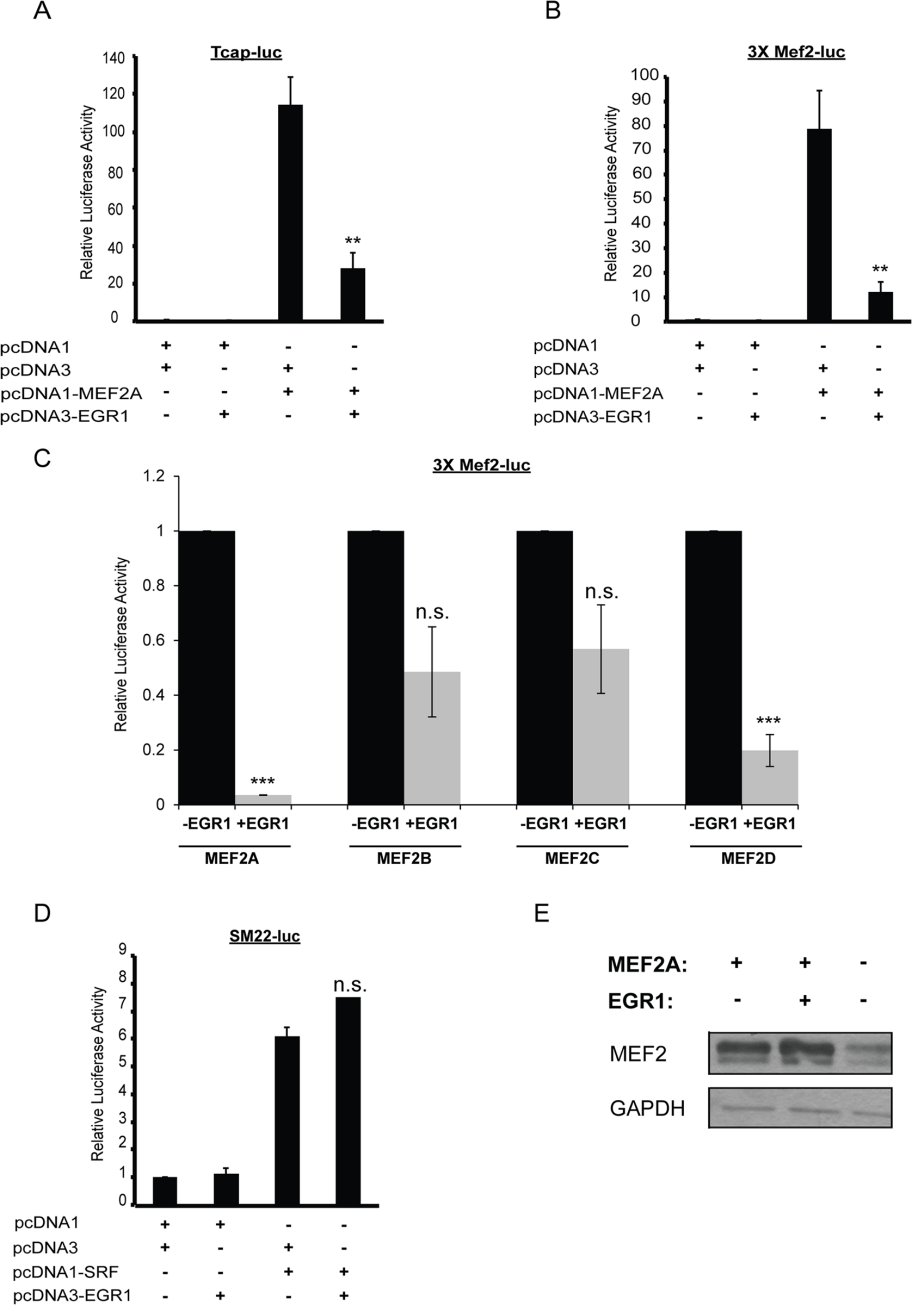
EGR1 is a potent repressor of MEF2 transcriptional activity. (A) EGR1 represses MEF2A transcriptional activity. HEK293T cells were transfected with the *Tcap*-Luc, pcDNA3-EGR1 and pcDNA1-MEF2A. pcDNA1 and pcDNA3 were used as empty plasmid controls for the MEF2A and EGR1 expression vectors, resepectively. Firefly luciferase readings were normalized by Bradford assay. (n = 6, p<0.003). (B) Western blot analysis shows that EGR1 overexpression does not decrease the expression of MEF2A. (C) HEK293T cells were transfected similarly to as in Panel A, but instead with the 3xMEF2-luc reporter vector. (n = 4, p<0.007). (D) EGR1 represses transcriptional activity of MEF2A and MEF2D, and there is a non-significant repressive trend of transcriptional activity by MEF2B and MEF2C. HEK293T cells were cotransfected with 3xMEF2-luc and MEF2A, B, C, or D in the presence or absence of EGR1. (E) EGR1 does not repress SRF activity. 293T cells were transfected with the luciferase reporter vector *SM22*-luc, EGR1 and SRF. Firefly luciferase readings were normalized by Bradford assay. No significant difference is seen in SRF activity with EGR1 (n = 4, n.s.). (F) MEF2A does not repress the activity of EGR1 on a EGR1-specific promoter construct, p300-luc, lacking MEF2 binding sites (n = 3).Data are mean ± SEM. *p<0.05, **p<0.01, ***p<0.001, n.s. not significant.

To investigate the possibility that EGR1 functions as a coregulator of MEF2A, we initially examined the effect of EGR1 on MEF2A transcriptional activity using the proximal promoter region of the mouse *Tcap* gene. The *Tcap* gene encodes a myofibrillar Z-disc protein associated with the costameric protein network whose expression in cardiomyocytes is directly regulated by MEF2A [4]. In addition to the MEF2 site, the 2.0 kilobase (kb) proximal promoter region of the mouse *Tcap* gene is predicted to harbor 5 EGR binding sites located at positions -127, -646, -1407, -1533, and -1851, relative to the transcription start site. HEK293T cells were co-transfected with the *Tcap*-luciferase reporter and MEF2A in the presence or absence of EGR1. As shown in Fig 1A, EGR1 alone had no significant effect on the basal activity of the *Tcap* reporter, whereas MEF2A robustly activated this reporter. In contrast, co-transfection of EGR1 significantly repressed MEF2A activation of the *Tcap* reporter. It is worth noting that this repressive effect was not due to EGR1 inhibiting the expression of the MEF2A expression plasmid as there was no decrease in overexpressed MEF2A in cells transfected with EGR1 (Fig 1B).

In a parallel series of experiments, we asked whether EGR1-mediated repression of MEF2A required DNA binding as previous studies have shown that EGR1 is able to repress NF-κB activity in a non-DNA binding manner [16]. Therefore, we examined the ability of EGR1 to repress a MEF2-dependent promoter not known to have EGR binding sites. For these experiments we used the 3xMEF2 reporter, which harbors three tandem copies of the MEF2 site from the *desmin* gene and flanking sequences that do not contain the consensus EGR DNA binding site [17]. The 3xMEF2 reporter was transfected in HEK293T cells along with MEF2A, EGR1, or both. EGR1 alone had no effect on the multimerized 3xMEF2 reporter but, similar to the *Tcap* promoter, significantly repressed this reporter in the presence of MEF2A (Fig 1C). These results suggest that EGR1 is capable of repressing MEF2A activity in a non-DNA binding fashion.

We next asked whether EGR1-mediated repression of MEF2A was restricted to this protein isoform or whether other MEF2 protein isoforms could also be inhibited by this factor. Although EGR1 was identified by analyzing the promoter regions of costamere associated genes regulated by MEF2A, we found that EGR1 also significantly repressed MEF2D transcriptional activity on the 3xMEF2 reporter (Fig 1D). EGR1 overexpression did not significantly repress transcriptional activity of MEF2B and MEF2C, but we noted a trend towards repression with these MEF2 isoforms (Fig 1D). These results indicate that, in addition to MEF2A, EGR1 is capable of significantly repressing the transcriptional activity of an additional MEF2 isoform.

To determine whether EGR1 specifically represses the MEF2 family or whether it is able to repress other members of the MADS-box transcription factor superfamily, we examined the effect of EGR1 on serum response factor (SRF) transcriptional activity. Like the MEF2 proteins, SRF possesses a MADS box DNA binding and dimerization domain but is unable to dimerize with these factors and it binds to a different A/T-rich consensus DNA binding sequence known as a CArG box [18]. HEK293T cells were co-transfected with SRF and the SRF-dependent *SM22*-luc reporter, with or without EGR1. As shown in Fig 1E, EGR1 failed to repress SRF activity on the *SM22*-luc reporter, demonstrating that EGR1 functions as a specific co-repressor of the MEF2 subclass of MADS-box transcription factors.

To investigate if this was a reciprocal repression of transcriptional activity, we transfected HEK293T cells with an EGR1-specific promoter, *p300*, reporter construct [19]. This promoter region lacks consensus MEF2 binding sites, and this promoter was selected to determine if MEF2A:EGR1 protein-protein interactions have similar effects on EGR1-activated genes. Unlike the MEF2-specific reporters, p300-luc was not repressed by the co-transduction of MEF2A and EGR1 (Fig 1F). These results demonstrate that the EGR1-mediated repressive effect on MEF2A transcriptional activity is not reciprocal and suggest that repression is specific for genes directly regulated by MEF2.

### EGR1 represses endogenous MEF2 transcriptional activity

**Fig 2:**
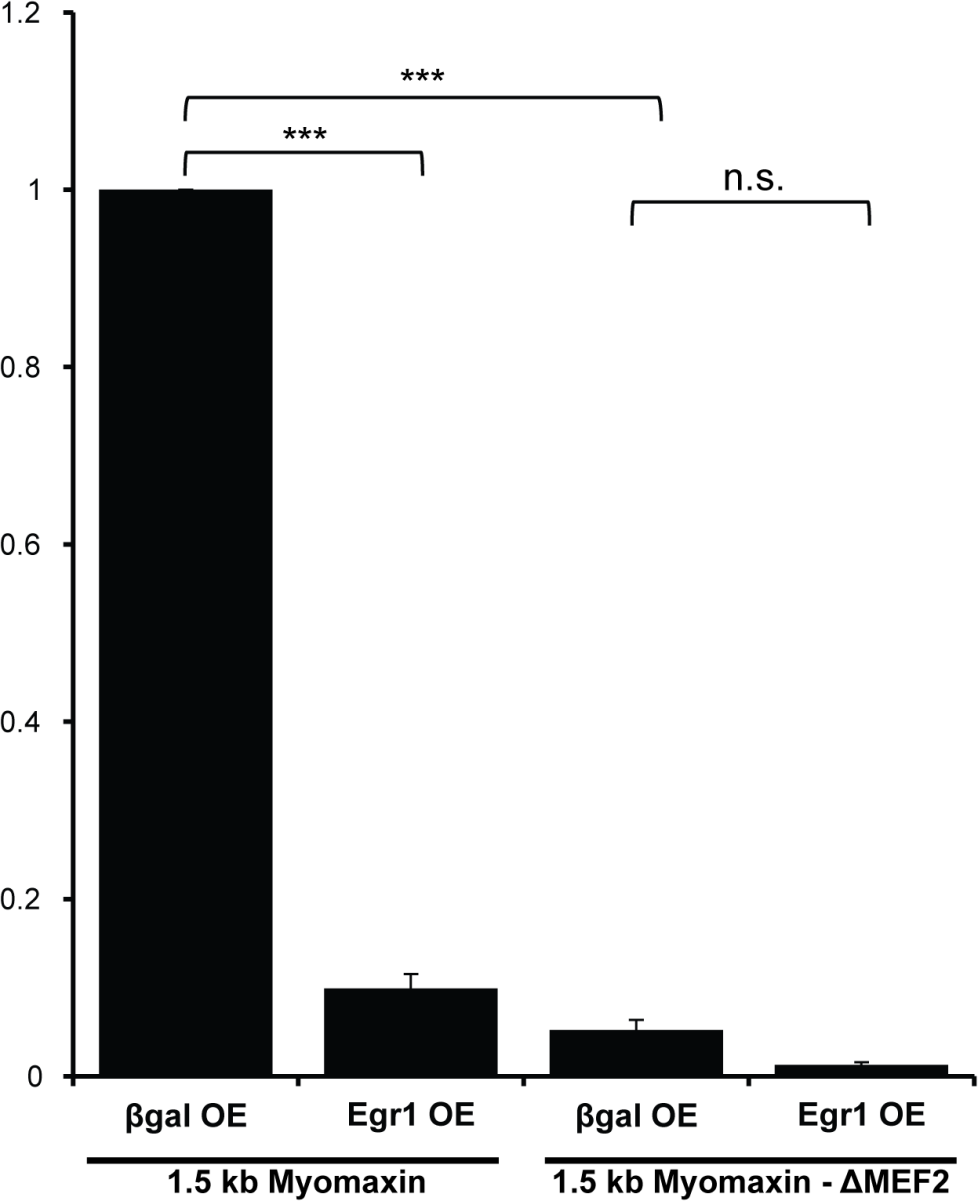
EGR1-mediated repression of endogenous MEF2 activity in cardiomyocytes is dependent on MEF2 DNA binding. Repression of MEF2-dependent costamere promoters by EGR1 requires intact MEF2 binding site. NRVMs were transduced with AdEGR1 and Adβgal at an MOI of 25. Twenty four hours post-transduction, 1.5 kb *myomaxin*-Luc and 1.5 kb *myomaxin*ΔMEF2-Luc were transfected. Luciferase activity was measured 48 hrs after transfection. Firefly luciferase readings were normalized to Renilla luciferase readings. Data are mean ± SEM. ***p<0.001, n.s. not significant.

To determine whether EGR1 represses endogenous MEF2 activity in cardiac muscle cells we examined the activity of MEF2-dependent reporters in neonatal rat ventricular myocytes (NRVMs) transduced with EGR1 adenovirus. Initially, we used the *Tcap* and 3xMEF2 reporters for these assays but their variable and low activities, respectively, in NRVMs precluded us from further analyzing the effect of EGR1 on these MEF2-dependent constructs. As an alternative, we used the 1.5 kb proximal promoter region of *myomaxin/Xirp2*, which has been shown to display consistently high MEF2-dependent activity in primary cardiomyocytes [20]. Additionally, similar to the 3xMEF2 reporter, the 1.5 kb *myomaxin* promoter region is predicted to lack EGR binding sites, thus any effect on reporter activity is likely to be mediated via DNA binding-independent effects of EGR1. As shown in Fig 2, overexpression of EGR1 significantly repressed the activity of the wild type 1.5 kb *myomaxin* promoter. In contrast, the mutant 1.5 kb *myomaxin* ΔMEF2 reporter, harboring a mutation in the -75 MEF2 site which results in reduced basal activity in NRVMs, was not significantly repressed by EGR1. These results reveal that EGR1-mediated repression of a MEF2 target gene in cardiomyocytes is primarily occurring through the inhibition of MEF2 activity.

### EGR1 interacts with MEF2A

The DNA-binding independent mechanism by which EGR1 represses MEF2-dependent transcription suggests that EGR1 represses MEF2 activity through protein-protein interaction. Therefore, a co-immunoprecipitation assay was performed to determine whether or not EGR1 and MEF2A interact in transfected cells.

**Fig 3:**
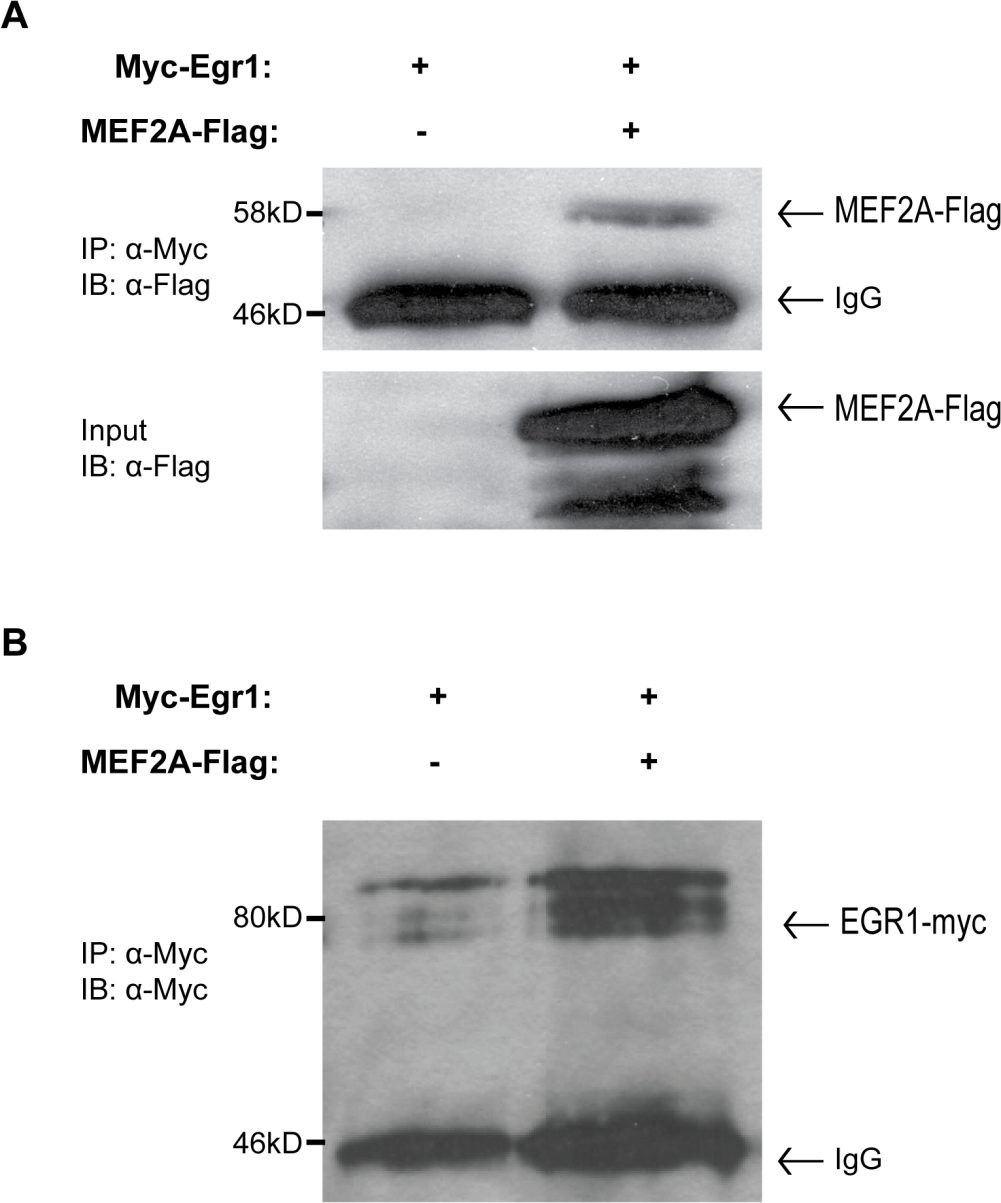
EGR1 and MEF2A interact *in vitro*. (A) HEK293T cells were transfected with pcDNA3-myc (empty vector) or pcDNA3-Myc-EGR1 (N-terminal epitope tag) and pCMV-MEF2A-FLAG (C-terminal epitope tag). Whole cell lysates in AT buffer were incubated with Protein G Sepharose Beads (GE Healthcare) and 1 μg of anti-Flag and incubated at 4°C, rotating overnight on a nutator. Precipitated samples were fractionated on an 8% SDS-PAGE gel followed by a western blot incubated with anti-Flag (1:2,000). (B) Self-immunoprecipitation of the myc-EGR1 protein shows efficient expression and purification.

Co-immunoprecipitation was performed using epitope-tagged fusion proteins MEF2A-FLAG (C-terminal tag) and MYC-EGR1 (N-terminal tag). To confirm the expression of these two epitope-tagged fusion proteins, MEF2A-Flag and EGR1were transfected into HEK293T cells and protein was harvested 36–48 hours post-transfection. Western blot analysis confirmed the expression of both MEF2A-FLAG (Fig 3A) as well as MYC-EGR1 (Fig 3B). Lysates from cells cotransfected with empty MYC vector or MYC-EGR1, and MEF2A-FLAG were then subjected to a co-immunoprecipitation assay to test for a protein-protein interaction. As shown in Fig 3A left panel, MEF2A was immunoprecipated effectively indicating an interaction between EGR1 and MEF2A. When empty MYC vector was immunoprecipitated, no MEF2A-FLAG was detectable (Fig 3A right panel), suggesting that the immunoprecipitation of MEF2A-FLAG was mediated by interaction with EGR1 rather than non-specific immunoprecipitation.

### Costamere gene expression and cardiomyocyte survival are sensitive to EGR1 expression

**Fig 4:**
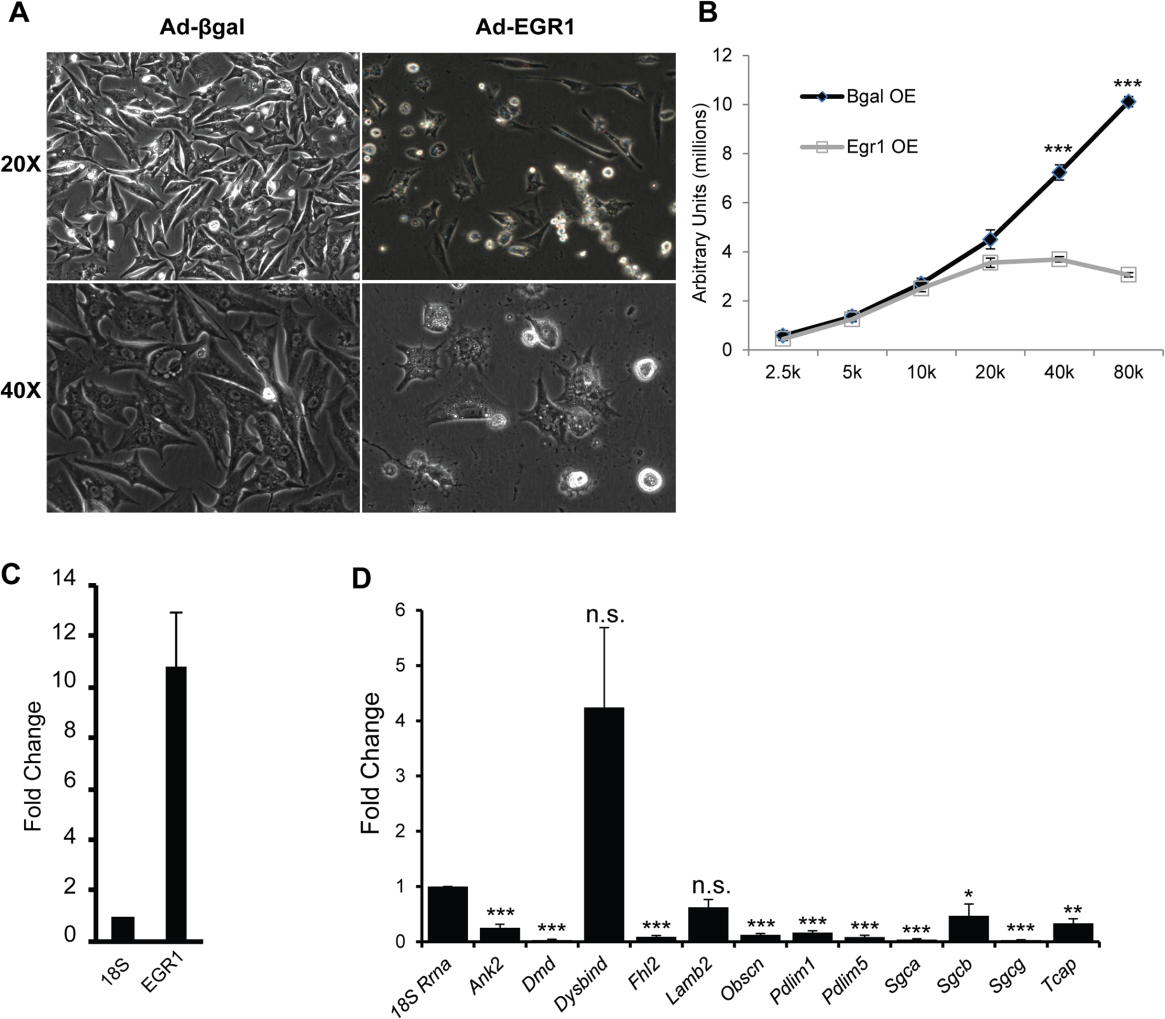
Costamere gene expression is sensitive to EGR1 levels in NRVMs. (A) qRT-PCR analysis confirms increased expression of Egr1 transcripts 48 hours post-transduction with AdEGR1; fold change is in comparison to expression levels in the Adβgal control, results were normalized to 18s. (B) NRVMs were transduced with AdEGR1 and Adβgal at an MOI of 25 and observed 48 hours post transduction. Extensive cell detachment is seen in the AdEGR1 transduced NRVMs in comparison to the Adβgal transduced NRVMs. (C) NRVMs were seeded in increasing cell densities and transduced with AdEGR1 or Adβgal at an MOI of 25 and assayed for cell viability 48 hours post-transduction. Cell titer blue assay shows a significant decrease in viability in AdEGR1 transduced NRVMs but not the control. (D) NRVMs were transduced with AdEGR1 or Adβgal at an MOI of 25 and assayed for Caspase 3 activity 72 hour post-transduction. The assay shows significant upregulation of Caspase 3 activity at 72 hours post-transduction in the AdEGR1-transduced, but not Adβgal-transduced NRVMs. (E) qRT-PCR analysis of 13 MEF2-dependent costamere genes shows 11 of these genes are down-regulated when EGR1 is overexpressed in NRVMs; fold change is in comparison to expression levels in the Adβgal control. Results were normalized to 18s. Data are mean ± SEM, n = 3, *p<0.05, **p<0.01, ***p<0.001.

EGR1 repression of MEF2 activity suggests that overexpression of this factor might impair cardiomyocyte survival in a manner similar to that observed in MEF2A-deficient NRVMs [4]. We investigated this hypothesis by transducing NRVMs with adenoviruses expressing either EGR1 (AdEGR1) or a beta-galactosidase (Adβgal) control. Quantitative RT-PCR analysis showed increased Egr1 expression in AdEGR1 treated NRVMs compared to Adβgal controls (Fig 4A). At 72 hours post transduction, we noted widespread cardiomyocyte detachment in EGR1-transduced NRVMs, similar to MEF2A-depleted NRVMs (Fig 4B, right panels). The cellular detachment phenotype suggested reduced viability of cardiomyocytes overexpressing EGR1. Analysis of cellular viability revealed significantly reduced survival in EGR1 expressing cells (Fig 4C). Measurement of Caspase 3 activity, a marker of apoptosis, showed a significant increase in Caspase 3 activity in EGR1 overexpressing cells, suggesting induction of apoptosis as a mechanism for the decreased viability of EGR1 overexpressing cell (Fig 4D). We next examined the expression of thirteen costamere genes previously characterized by our lab to be downregulated in MEF2A-depleted NRVMs [4]. Consistent with the ability of EGR1 to repress MEF2 activity, EGR1 overexpression in NRVMs led to significantly decreased expression of eleven of the thirteen MEF2-dependent costamere genes analyzed (Fig 4E).

**Fig 5:**
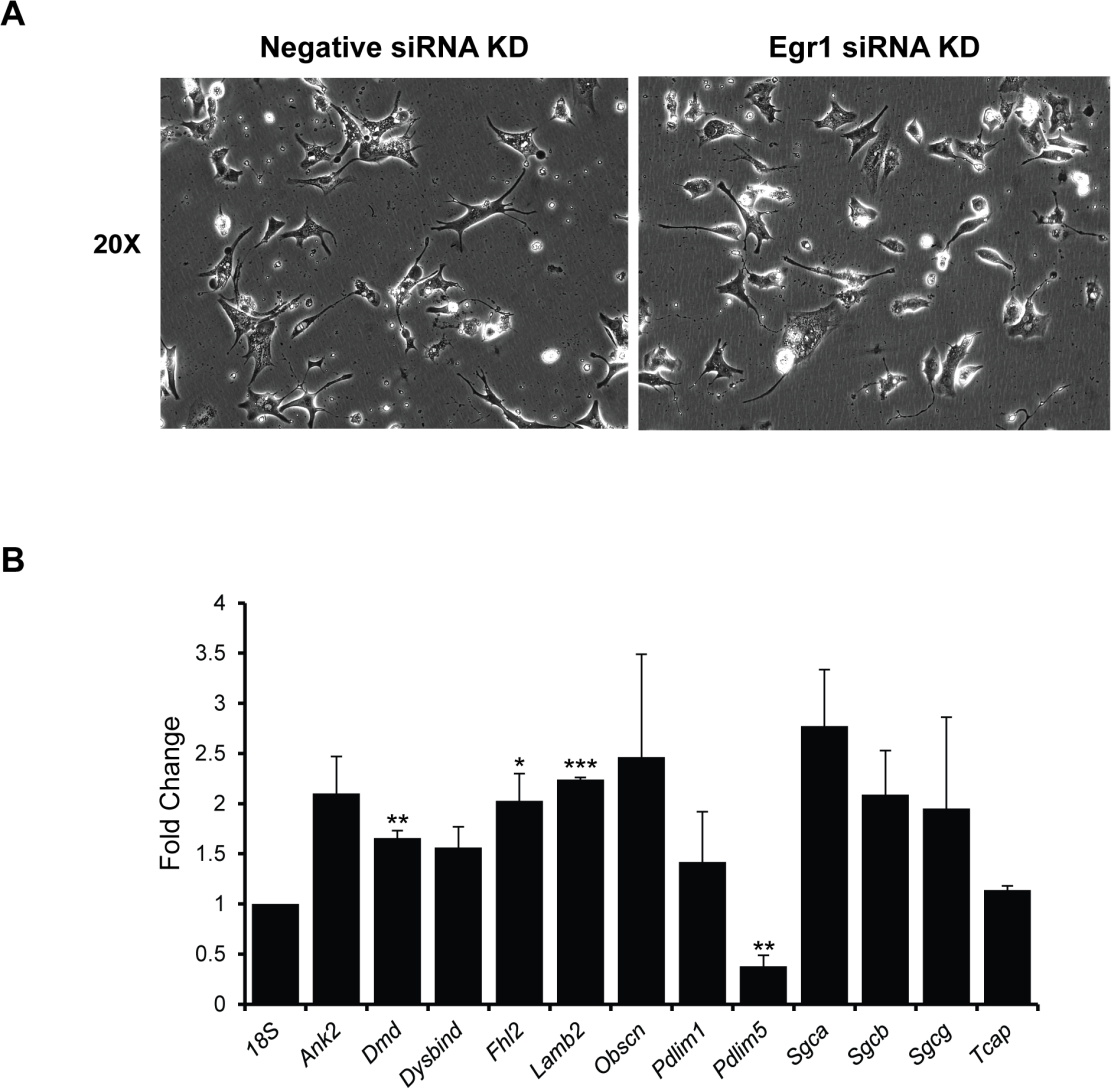
Costamere gene expression is upregulated in EGR1-depleted NRVMs. (A) EGR1 depleted NRVMs do not display any obvious morphological defects. NRVMs were transfected with 100 nM EGR1 siRNA and analyzed 72 hours post transfection. (B) HEK 293T cells were transfected with pcCMV-EGR1-Flag, and either a negative control or Egr1 siRNA. Western blot analysis probing for the Flag epitope shows loss of EGR1-Flag expression upon co-transfection with the Egr1 siRNA, though a confounding non-specific band is present slightly above the EGR1-Flag band. (C) EGR1-depletion results in upregulated costamere gene expression in NRVMs. qRT-PCR analysis of 13 MEF2-dependent costamere genes in EGR1 siRNA knockdown NRVMs shows that eight of the genes are significantly upregulated, and the majority of the remaining genes show a nonsignificant trend towards upregulationwhen EGR1 is knocked down; fold change is in comparison to expression levels in the negative siRNA knockdown controls, results were normalized to 18s. Sample size for Ank2, Dmd, Dysbind, Lamb2, Pdlim1, Sgcb, and 18s is n = 6. Sample size for Pdlim5, Sgca, Sgcg, and Tcap is n = 5. Sample size for Fhl2 and Obscn is n = 4, and the sample size for Xirp2 is n = 3., *p<0.05, ** p<0.01, ***p<0.001.

In a complementary set of experiments EGR1 was depleted in NRVMs using an EGR1-specific siRNA (Ambion). Unlike EGR1 overexpression, siRNA-mediated knockdown of EGR1 had no obvious morphological effect on NRVMs 72 hrs post-transfection (Fig 5A). To determine the efficiency of EGR1 knockdown, HEK293T cells were transfected with pcDNA3-EGR1-Flag with either negative control siRNA or Egr1 siRNA. Cell lysates were probed for Flag expression and EGR1-Flag expression is observed at the expected size (80kD) when cotransfected with negative control siRNA, but expression is lost when cotransfected with Egr1 siRNA. There is a confounding non-specific band just above the relevant EGR1-Flag band that is observed in all samples, including the untransfected control, and does not vary with siRNA treatment. Subsequently, expression of costamere genes was analyzed by qRT-PCR. As predicted by our model of EGR1-mediated MEF2 repression, MEF2-dependent costamere gene expression was elevated in EGR1 siRNA knockdown NRVMs (Fig 5C). Eight of the thirteen genes were significantly upregulated upon Egr1 knockdown. Taken together, the EGR1 over- and under-expression experiments clearly support its role in MEF2-dependent costamere gene expression in cardiomyocytes.

## Discussion

The present report reveals that the EGR1 transcription factor interacts with MEF2A and functions as a potent repressor of MEF2 activity in cardiomyocytes. EGR1 was shown to repress MEF2A transcriptional activity on MEF2-dependent promoters that either harbored multiple, predicted EGR binding sites or lacked a consensus EGR DNA binding sequence. Moreover, although we previously identified EGR1 through computational analysis of predicted transcription factor binding sites on costamere genes primarily dependent on MEF2A, our investigation revealed that EGR1 significantly represses additional MEF2 protein isoforms, primarily MEF2D, in transient reporter assays. Finally, this repressive effect was found to be specific for the MEF2 subclass of MADS box transcription factors as EGR1 failed to repress SRF transcriptional activity.

While EGR1 was selected as a candidate coregulator of MEF2A through analysis of consensus DNA binding sequences on a collection of costamere promoters, it is interesting that the repressive effect of EGR1 could also occur in a DNA-binding independent manner. This suggests that the role of DNA-binding, as it relates to the repressive action of EGR1, may be to more effectively target the EGR1 protein to promoters that are directly bound by MEF2. It is unclear whether EGR1 DNA-binding plays an additional non-MEF2 related role in the regulation of cardiomyocyte costamere and survival, and further study is required to better understand how the repressive interaction of EGR1 and MEF2 is reprised in a physiological context.

It is interesting that EGR1 significantly repressed the activity of MEF2A and D but not MEF2B or C. All MEF2 cofactor interactions to date have failed to reveal isoform selective differences in modulating their transcriptional activity. While it is difficult to make firm conclusions based on heterologous reporter assays, and additional molecular dissection of these differences is clearly required, it is tempting to speculate that MEF2A and D share biochemical properties not present in either MEF2B or C. For example, comparison of the four murine MEF2 protein sequences revealed two small polypeptide regions of high similarity in MEF2A and MEF2D [SLV(S/T)PSL(A/V)A(S/T)S and MPTAYNTDY] which are not conserved in the other MEF2 protein isoforms. Alternatively, the isoform selectivity may be related to encoded functional differences in transcriptional activity given that MEF2A and MEF2D are the predominant isoforms expressed in postnatal cardiomyocytes.

Consistent with the notion that EGR1 repressed MEF2, overexpression of EGR1 in NRVMs resulted in significantly decreased expression of MEF2-dependent costamere genes. Interestingly, overexpression of EGR1 also caused cardiomyocyte detachment and significantly reduced survival, which is reminiscent of the MEF2A depletion phenotype in NRVMs [4]. Conversely, EGR1 knockdown resulted in significant upregulation of eight of the thirteen costamere genes. However, these effects were rather modest suggesting that other EGR family members partially compensate for EGR1. These observations support our model that EGR1 and MEF2 function in the same pathway to transcriptionally coregulate MEF2-dependent costamere genes in cardiomyocytes. Moreover, although our data suggest that EGR1 directly represses MEF2 activity it is plausible that histone deacetylase (HDAC) recruitment may participate in the repressive mechanism, particularly given the involvement of these chromatin repressor proteins in the regulation of MEF2 activity [21]. Along these lines, EGR1 transcriptional activity has been shown to be modulated by HDAC4, raising the possibility that EGR1 facilitates the targeting of HDACs to MEF2-regulated costamere promoters [22]. But unlike that report, which described enhanced activity on HDAC4 recruitment, in the context of costamere gene regulation would result in repression of these genes in cardiac muscle.

The ability of EGR1 to function as a repressor of gene expression in cardiomyocytes has been documented [23,24]. However, EGR1 has not been previously demonstrated to regulate a distinct gene program or specifically interact with and repress the activity of a core cardiac transcription factor such as MEF2. Of particular interest is the connection of these transcription factors in cardiac hypertrophy pathways. MEF2 is a known downstream mediator of hypertrophic signaling [25], particularly in calcineurin-induced hypertrophy by Ca^2+^-activated NFAT signaling that promotes chamber dilation and loss of contractility [26]. In this regard, EGR1 has been shown to induce Cav3.2 T-type calcium channels, which plays a role in inducing calcineurin/NFAT signaling during cardiac hypertrophy [27], to play an important role in adaptive response to hypertrophic stimuli [28], and to be targeted and suppressed by Atf3during endothelin-1 induced cardiomyocyte hypertrophy [29]. Additionally, Nab1, a repressor of EGR, has been shown to be a potent inhibitor of pathological cardiac hypertrophy [30] and EGR1 deficient mice have a blunted catecholamine-induced hypertrophy response and are more sensitive to stress [31]. Given the wide variety of cardiopathologies in which both EGR1 and MEF2 have been implicated, further investigation of EGR1’s function as a potent MEF2 repressor will provide additional insight into mechanisms of various cardiopathologies and into potential targets for treatment of cardiac disease.
