# Correction: EGR1 Functions as a Potent Repressor of MEF2 Transcriptional Activity

**DOI:** 10.1371/journal.pone.0131619

**Published:** 2015-06-24

**Authors:** Yi Feng, Cody A. Desjardins, Olivia Cooper, Akuah Kontor, Sarah E. Nocco, Francisco J. Naya

The images for Figs 1, 3, 4, and 5 are previous versions of the figures. Please see the correct figures here.

**Fig 1:**
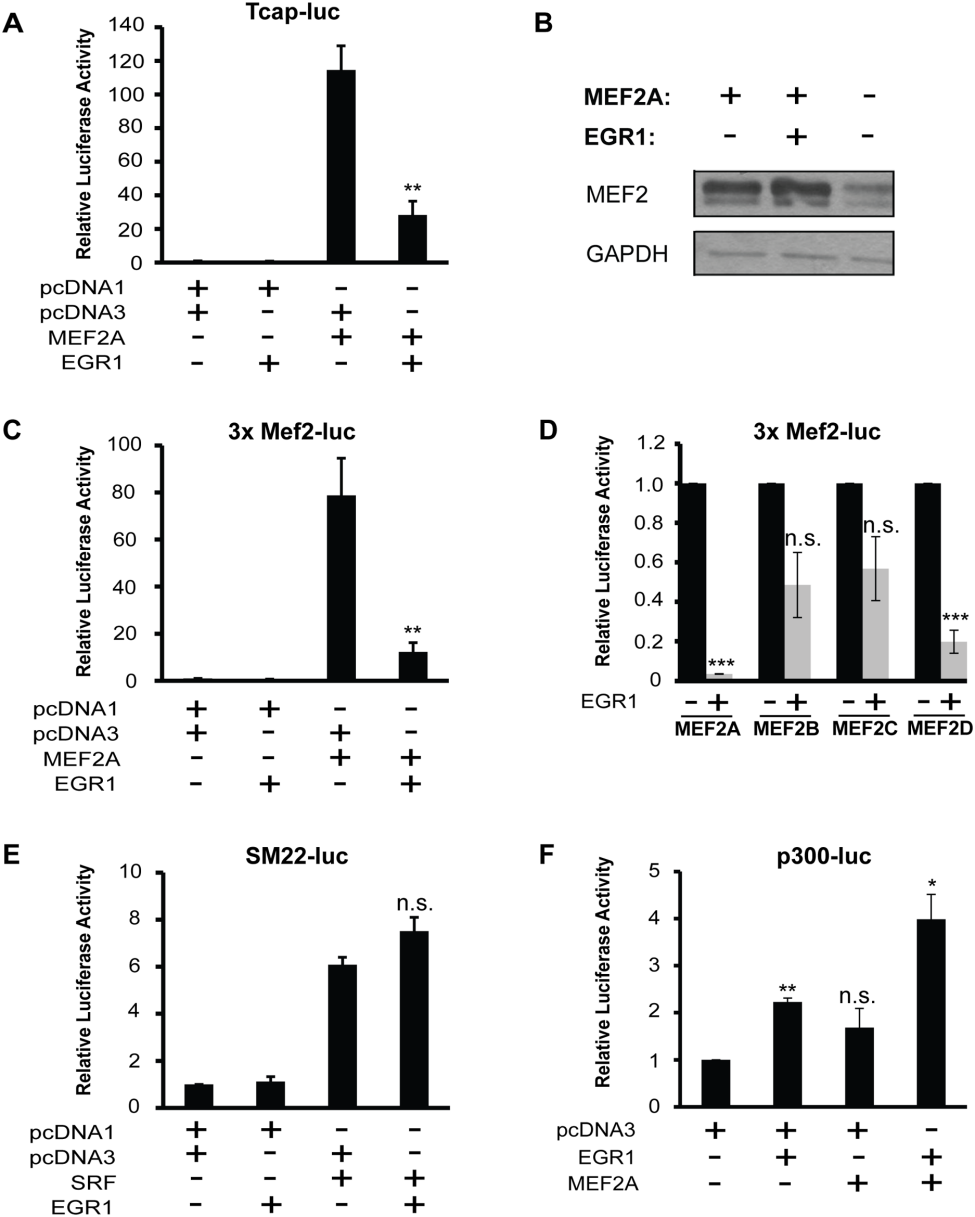
EGR1 is a potent repressor of MEF2 transcriptional activity. (A) EGR1 represses MEF2A transcriptional activity. HEK293T cells were transfected with the *Tcap*-Luc, pcDNA3-EGR1 and pcDNA1-MEF2A. pcDNA1 and pcDNA3 were used as empty plasmid controls for the MEF2A and EGR1 expression vectors, resepectively. Firefly luciferase readings were normalized by Bradford assay. (n = 6, p<0.003). (B) Western blot analysis shows that EGR1 overexpression does not decrease the expression of MEF2A. (C) HEK293T cells were transfected similarly to as in Panel A, but instead with the 3xMEF2-luc reporter vector. (n = 4, p<0.007). (D) EGR1 represses transcriptional activity of MEF2A and MEF2D, and there is a non-significant repressive trend of transcriptional activity by MEF2B and MEF2C. HEK293T cells were cotransfected with 3xMEF2-luc and MEF2A, B, C, or D in the presence or absence of EGR1. (E) EGR1 does not repress SRF activity. 293T cells were transfected with the luciferase reporter vector *SM22*-luc, EGR1 and SRF. Firefly luciferase readings were normalized by Bradford assay. No significant difference is seen in SRF activity with EGR1 (n = 4, n.s.). (F) MEF2A does not repress the activity of EGR1 on a EGR1-specific promoter construct, p300-luc, lacking MEF2 binding sites (n = 3). Data are mean ± SEM. *p<0.05, **p<0.01, ***p<0.001, n.s. not significant.

**Fig 3:**
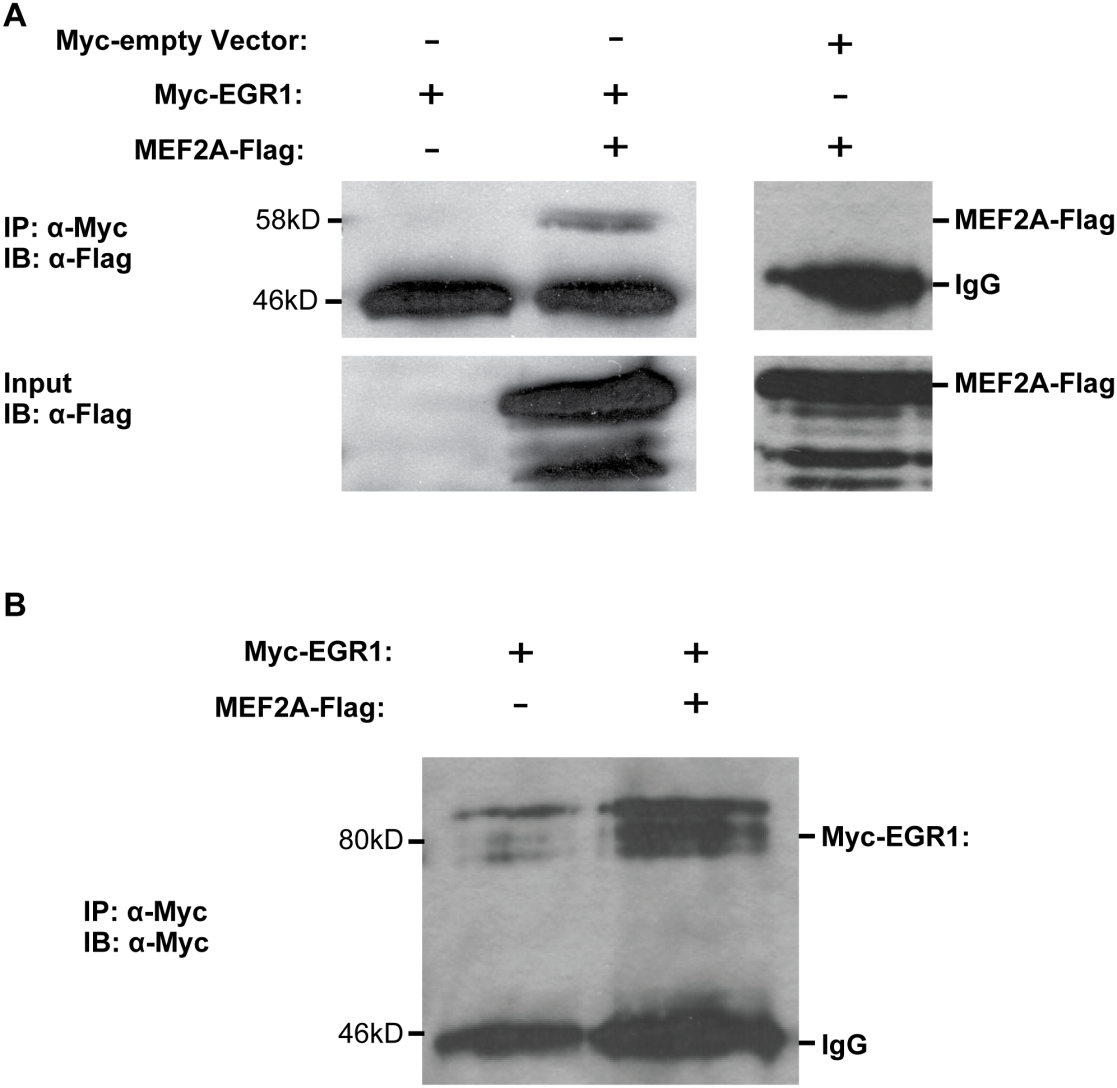
EGR1 and MEF2A interact in vitro. (A) HEK293T cells were transfected with pcDNA3-myc (empty vector) or pcDNA3-Myc-EGR1 (N-terminal epitope tag) and pCMV-MEF2A-FLAG (C-terminal epitope tag). Whole cell lysates in AT buffer were incubated with Protein G Sepharose Beads (GE Healthcare) and 1 μg of anti-Flag and incubated at 4°C, rotating overnight on a nutator. Precipitated samples were fractionated on an 8% SDS-PAGE gel followed by a western blot incubated with anti-Flag (1:2,000). (B) Self-immunoprecipitation of the myc-EGR1 protein shows efficient expression and purification.

**Fig 4:**
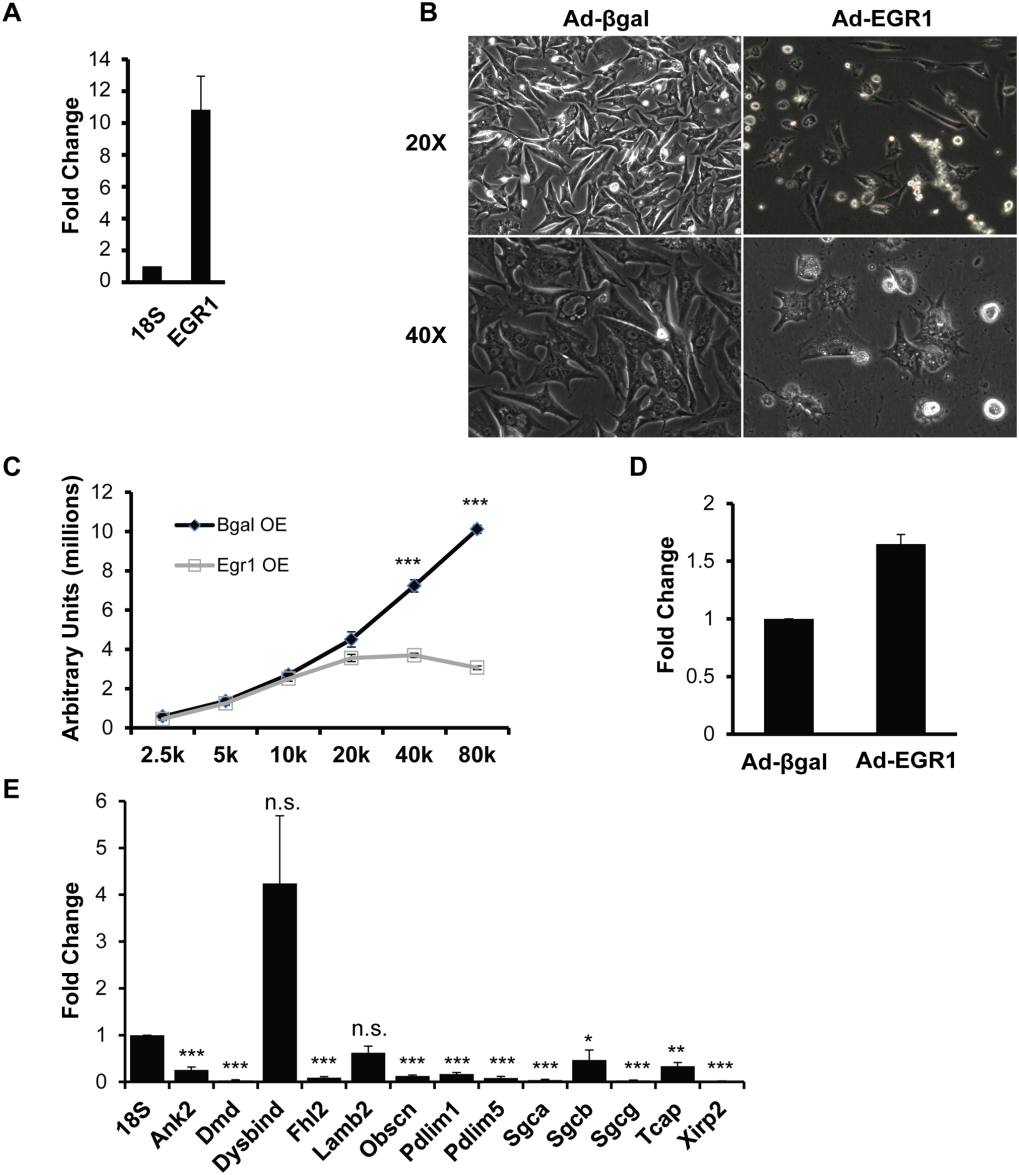
Costamere gene expression is sensitive to EGR1 levels in NRVMs. (A) qRT-PCR analysis confirms increased expression of Egr1 transcripts 48 hours post-transduction with AdEGR1; fold change is in comparison to expression levels in the Adβgal control, results were normalized to 18s. (B) NRVMs were transduced with AdEGR1 and Adβgal at an MOI of 25 and observed 48 hours post transduction. Extensive cell detachment is seen in the AdEGR1 transduced NRVMs in comparison to the Adβgal transduced NRVMs. (C) NRVMs were seeded in increasing cell densities and transduced with AdEGR1 or Adβgal at an MOI of 25 and assayed for cell viability 48 hours post-transduction. Cell titer blue assay shows a significant decrease in viability in AdEGR1 transduced NRVMs but not the control. (D) NRVMs were transduced with AdEGR1 or Adβgal at an MOI of 25 and assayed for Caspase 3 activity 72 hour post-transduction. The assay shows significant upregulation of Caspase 3 activity at 72 hours post-transduction in the AdEGR1-transduced, but not Adβgal-transduced NRVMs. (E) qRT-PCR analysis of 13 MEF2-dependent costamere genes shows 11 of these genes are down-regulated when EGR1 is overexpressed in NRVMs; fold change is in comparison to expression levels in the Adβgal control. Results were normalized to 18s. Data are mean ± SEM, n = 3, *p<0.05, **p<0.01, ***p<0.001.

**Fig 5:**
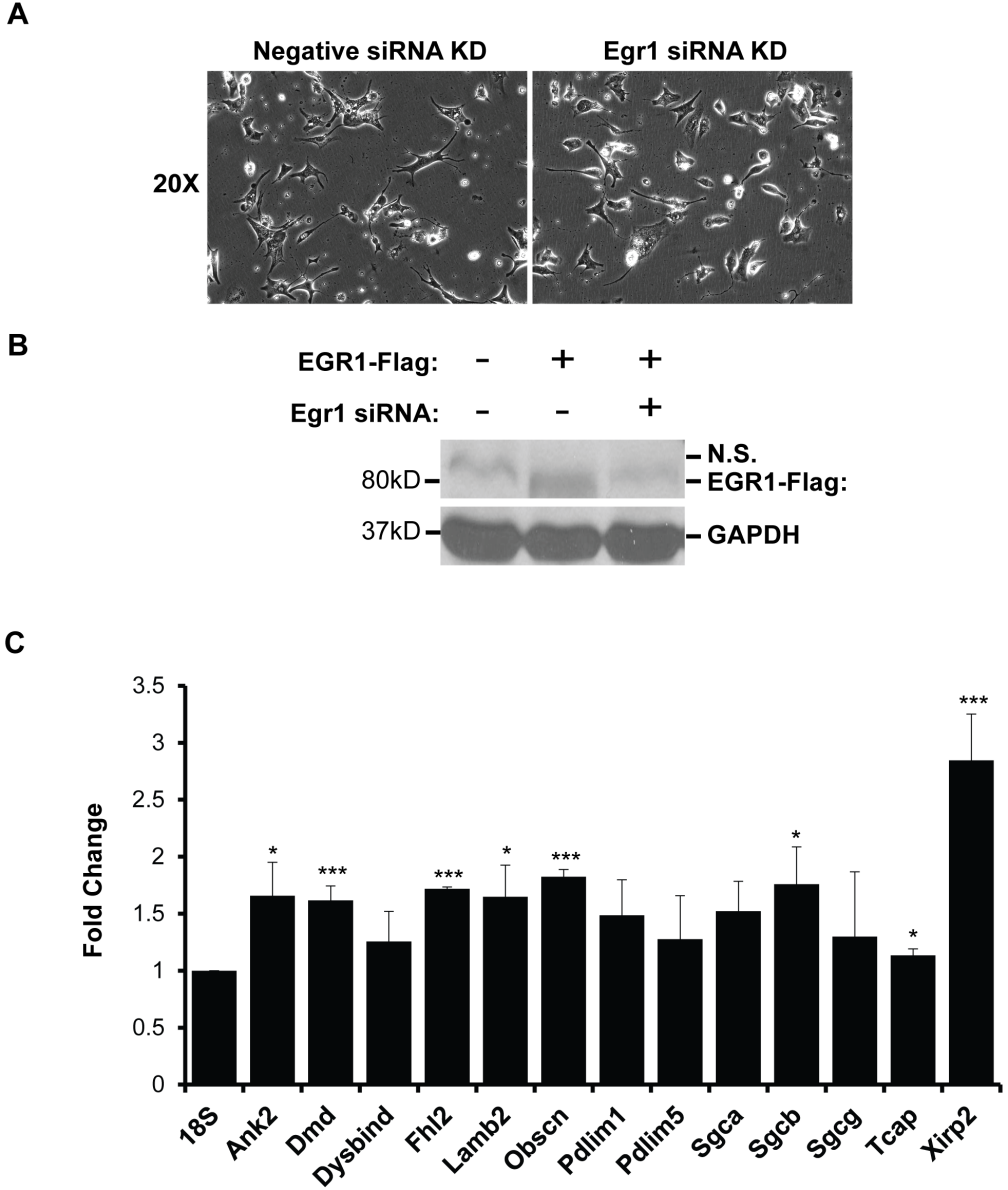
Costamere gene expression is upregulated in EGR1-depleted NRVMs. (A) EGR1 depleted NRVMs do not display any obvious morphological defects. NRVMs were transfected with 100 nM EGR1 siRNA and analyzed 72 hours post transfection. (B) HEK 293T cells were transfected with pcCMV-EGR1-Flag, and either a negative control or Egr1 siRNA. Western blot analysis probing for the Flag epitope shows loss of EGR1-Flag expression upon co-transfection with the Egr1 siRNA, though a confounding non-specific band is present slightly above the EGR1-Flag band. (C) EGR1-depletion results in upregulated costamere gene expression in NRVMs. qRT-PCR analysis of 13 MEF2-dependent costamere genes in EGR1 siRNA knockdown NRVMs shows that eight of the genes are significantly upregulated, and the majority of the remaining genes show a nonsignificant trend towards upregulation when EGR1 is knocked down; fold change is in comparison to expression levels in the negative siRNA knockdown controls, results were normalized to 18s. Sample size for Ank2, Dmd, Dysbind, Lamb2, Pdlim1, Sgcb, and 18s is n = 6. Sample size for Pdlim5, Sgca, Sgcg, and Tcap is n = 5. Sample size for Fhl2 and Obscn is n = 4, and the sample size for Xirp2 is n = 3., *p<0.05, ** p<0.01, ***p<0.001.
